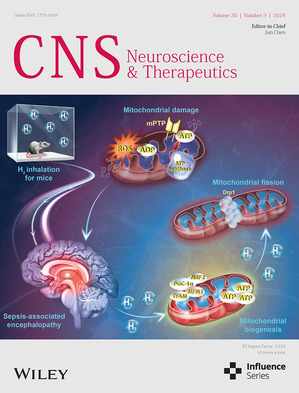# Front Cover

**DOI:** 10.1111/cns.70072

**Published:** 2024-09-26

## Abstract

The cover image is based on the article *High‐concentration hydrogen inhalation mitigates sepsis‐associated encephalopathy in mice by improving mitochondrial dynamics* by Yan Cui et al., https://doi.org/10.1111/cns.70021.